# From Nonalcoholic Fatty Liver Disease (NAFLD) to Metabolic Dysfunction-Associated Fatty Liver Disease (MAFLD)—New Terminology in Pediatric Patients as a Step in Good Scientific Direction?

**DOI:** 10.3390/jcm10050924

**Published:** 2021-03-01

**Authors:** Marta Flisiak-Jackiewicz, Anna Bobrus-Chociej, Natalia Wasilewska, Dariusz Marek Lebensztejn

**Affiliations:** Department of Pediatrics, Gastroenterology, Hepatology, Nutrition and Allergology, Medical University of Bialystok, 15-274 Bialystok, Poland; anna.chociej@op.pl (A.B.-C.); nwasilewska@interia.pl (N.W.); lebensztejn@hoga.pl (D.M.L.)

**Keywords:** MAFLD, NAFLD, fatty liver, metabolic syndrome, obesity, children, nomenclature

## Abstract

Nonalcoholic fatty liver disease (NAFLD) is the most common chronic liver disease in the world, which predispose to more serious hepatic conditions. It ranges from simple liver steatosis to nonalcoholic steatohepatitis (NASH), which may progress to cirrhosis, and even end-stage liver disease. Since obesity became one of the most important health concerns wordwide, a considerable increase in the prevalance of NAFLD and other metabolic implications has been observed, both in adults and children. Due to the coexistence of visceral obesity, insulin resistance, dyslipidemia, NAFLD is considered to be the hepatic manifestation of metabolic syndrome (MetS). These relationships between NAFLD and MetS led to the set up in adults of a new term combining both of these conditions, called metabolic dysfunction-associated fatty liver disease (MAFLD). Based on these findings, we propose a set of criteria, which may be useful to diagnose MAFLD in children and adolescents.

## 1. Introduction

As shown by previous research, nonalcoholic fatty liver disease (NAFLD) is one of the most important causes of liver pathology worldwide, and in the coming decades it will probably become the leading cause of end-stage liver disease. It is regarded as a spectrum of hepatic conditions, which ranges from simple steatosis without specific inflammatory changes, through to more severe nonalcoholic steatohepatitis (NASH), referring to hepatocyte ballooning degeneration with or without fibrosis, which may progress to cirrhosis with all its consequences, e.g., increased risk of developing hepatocellular carcinoma (HCC) and hepatic decompensation [1,2,3]. The prevalence of NAFLD ranges from 9–37% in the general population and has significantly increased over the last two decades. The discrepancy in the prevalence of NAFLD among studies is most likely due to differences in diagnostic modalities, accepted standards for the laboratory tests, diagnostic criteria, as well as dietary and lifestyle habits in different regions of the world [4]. A particularly worrying phenomenon is the rising prevalence of NAFLD observed among children and adolescents, which is an effect of globally dramatic dimensions of obesity, remaining one of the most challenging problems in western civilization. The authors of a meta-analysis from 2015, based on 74 clinical trials, estimated the prevalence of NAFLD in obese children and adolescents at 34.2%, whereas in the general pediatric population it was 7.6% [5]. Unfortunately, it may be associated with early unfavorable metabolic complications and the development of more serious liver conditions in which liver transplantation is the only available treatment option [6].

Based on current knowledge, the pathogenesis of NAFLD is a multifactorial and multi-step process. NAFLD is a clinicopathologic condition characterized by abnormal lipid deposition in the liver defined as steatosis of >5% of hepatocytes in liver biopsy or fat fraction > 5.6% assessed by proton magnetic resonance spectroscopy (^1^HMRS), in the absence of secondary causes of liver injury and excessive alcohol consumption. In children, the diagnostic criteria for NAFLD include steatosis in ultrasonography and abnormal liver tests with exclusion of other liver diseases. Liver biopsy is considered the gold-standard in NAFLD diagnosis, but it is not performed routinely due to its invasiveness and high cost. Indications for liver biopsy include uncertain diagnosis, suspected advanced liver disease or before treatment [7].

It is widely accepted that NAFLD is pathogenically a “multiple-hit” disease and a combination of genetic predisposition, the role of lipotoxicity, adipocytokines, altered gut-derived microbiome, as well as environmental factors like high-fat diet, excessive fructose consumption, sleep deprivation, or sedentary lifestyle [8,9,10,11]. Among established clinical conditions closely associated with NAFLD, particular attention is paid to abdominal obesity and other features of the metabolic syndrome (MetS) [12,13,14], including disturbed glucose metabolism with insulin resistance (IR) [15,16,17], dyslipidemia [18,19,20], hypertension [21], and other metabolic disorders connected with increase cardiovascular risk [22]. Due to this connection, NAFLD is widely considered as hepatic manifestation of MetS [23]. The pathogenetic mechanisms explaining the relationships between NAFLD and MetS are not fully understood, however visceral obesity, insulin resistance, and subclinical inflammation seem to play the key role in developing both diseases. This strict connection between hepatic steatosis and metabolic dysfunctions led to the creation of a new term for fatty liver accompanied by other components of MetS in adults called metabolic dysfunction-associated fatty liver disease (MAFLD). Therefore, the question arises whether proposed terminology should also be appropriately changed in children and adolescents?

## 2. Definition of Metabolic Syndrome in Children and Adolescents

MetS is an essential health problem, that involves a group of factors that together increase a risk of cardiovascular disease (CVD) [24,25] and is associated with IR and type 2 diabetes mellitus (T2DM) [26,27]. It seems that MetS risk factors can appear at any stage of life from childhood to adulthood, that is why there is a necessity to urgently identify children at increased risk for cardiometabolic comorbidity as early in life as possible, to enable them to initiate preventive treatment before concomitant disease has occurred.

To assess cardiometabolic risk in obese children and adolescents it is important to perform a detailed clinical examination with evaluation of anthropometric measures, such as height, weight, waist, and hip circumferences, and applying age- and gender-specific centiles. Many studies have shown that waist circumference (WC), waist-hip ratio (WHR), or waist-to-height-ratio (WHtR) may be useful and simple tools for central obesity screening, as well as cardiovascular risk and NAFLD assessment in both children and adults [28,29,30]. The degree of overweight-ness or obesity could be determined based on the calculation of body mass index (BMI), which should be referred to appropriate centiles curves [31]. In a population-based study, it has been shown that adolescents with a BMI ≥ 99th percentile have a significantly greater risk for having cardiovascular risk factor clustering compared to those with lower degrees of obesity [32]. However, we should be careful in making this kind of conclusions, because generally BMI is not an accurate parameter for the assessment health condition in an obese person. WC definitely seems to be better, which is considered as an important measurement correlated with visceral fat accumulation. According to the data from the Bogolusa Heart Study higher cardiometabolic risk and prevalence of MetS were found among normal weight centrally obese children (WHtR ≥ 0.5) than in overweight or obese children without abdominal obesity (WHtR < 0.5) [33].

While the definition of MetS in adults is well-established, it is more problematic in children because of a multitude of criteria with various settings of cut-off values, as well as difficulties in predicting future risk for developing CVD and T2DM. Reinehr et al. compared different MetS definitions in a cohort of 1205 children and adolescents to establish the prevalence of MetS. Researchers emphasized that it differed significantly depending on the definition criteria applied and ranged from 6 to 39% [34]. Some authors suggest that the prevalence of individual components of MetS in the pediatric population would be more relevant for the assessment of cardiovascular risk than the diagnosis of MetS [35]. In 2005, Alberti et al. proposed a global definition for MetS in adults. Diagnostic criteria for MetS in adults include central obesity (increased waist circumference ≥ 94 cm for males and ≥80 cm for females) and the presence of at least two additional abnormalities: dyslipidemia (increased triglycerides concentration (≥1.7 mmol/L (150 mg/dl) or reduced high-density lipoprotein cholesterol (HDL-C) concentration (<1.03 mmol/L (40 mg/dl) in males and <1.29 mmol/L (50 mg/dl) in females); or specific treatment for these lipid abnormalities), high blood pressure (systolic: ≥130 mmHg or diastolic: ≥85 mmHg or treatment of previously diagnosed hypertension) and glucose intolerance (fasting plasma glucose ≥ 5.6 mmol/L (100 mg/dl) or previously diagnosed 2TDM) [36]. Then, two years later, a new definition from the International Diabetes Federation (IDF) was developed that included all previous studies [37]. According to the presented criteria in children older than 16 years, the IDF adult criteria may be used for MetS diagnosis. In the group of patients aged 10–16 years, diagnosing the MetS requires the presence of abdominal obesity diagnosed based on age- and gender-specific percentile curves of WC (≥90th percentile) and two or more other following metabolic factors: hypertriglyceridemia, low HDL-C, high blood pressure, or glucose intolerance. In the youngest children between 6 and 10 years old, MetS cannot be diagnosed, but if the child’s waist circumference is over or equal to the 90th percentile further measurements should be made especially if there is a family history of metabolic disturbances. It is worth noting that in recent years, special attention has been paid to a certain role of low-grade systemic inflammation in the pathogenesis of MetS. Therefore, numerous studies have been carried out to uncover how inflammation contributes to the development and progression of MetS [38,39]. On the basis of new research it seems that evaluation of high sensitivity C-reactive protein (hs-CRP) could be useful in prediction of MetS, however this parameter is not in current diagnostic MetS criteria [40,41]. However, one of the most important limitations of the proposed MetS definition in children and adolescents is not taking into consideration NAFLD, hyperuricemia or sleep apnea, which are significantly associated with increased cardiometabolic risk in later life. Furthermore, it has been described that many of the metabolic and cardiovascular complications of obesity were already detectable in prepubertal children [42]. Therefore, MetS definition should be extended to prepubertal children, which is not currently the case. Due to the possible early onset of metabolic disorders, early prevention of MetS during childhood might not only decrease chronic disease burden early in life but also lower the proportion of adults who will develop cardiometabolic disease. Appropriate therapy and lifestyle change in these patients and their families will help to prevent the negative effects of obesity in the future.

## 3. Pathogenetic Mechanisms Linking NAFLD and MetS in Obese Children, Adolescents, and Adults

**Figure 1:**
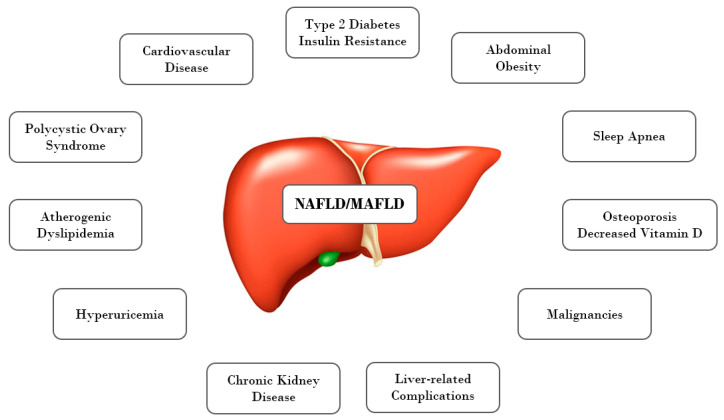
NAFLD/MAFLD: a multi-organ disorder. NAFLD: Nonalcoholic fatty liver disease. MAFLD: metabolic dysfunction-associated fatty liver disease.

The relation between NAFLD and MetS is one of the most often discussed problems. Numerous studies have shown a link between them, as well as a strong association of NAFLD in obese children with multiple cardiovascular risk factors (Figure 1). The presence of metabolic disorders such as T2DM, obesity, dyslipidemia, and hypertension carries a high risk of disease progression and development of NASH and fibrosis in NAFLD patients [43,44]. The estimated prevalence of MetS was approximately 18% of non-obese and 67% of obese individuals with NAFLD [2].

The study conducted in overweight and obese children presenting with hepatomegaly or elevated alanine aminotransferase (ALT) has revealed that patients labeled as having MetS had significantly higher risk to have NAFLD proven by biopsy than those who did not meet the criteria of MetS. However, they did not demonstrate a difference in ALT activity or echogenicity of the liver by ultrasound examination (USG) [45]. Yi-Wen Ting et al. have proven that obese children with MetS are more likely to have advanced liver fibrosis measured by transient elastography (using Fibroscan). Moreover, metabolic components such as BMI, WC, systolic blood pressure (SBP), serum triglycerides (TG) levels, serum total cholesterol (TC) level, hemoglobin A1c (HbA1c), 2TDM, and Homeostatic Model Assessment for Insulin Resistance (HOMA-IR) were significantly higher in patients with liver fibrosis. Multivariate regression analysis shown that waist circumference is a significant independent predictor of liver fibrosis [46]. Analyses of one of the studies, which was conducted in a large cohort of obese adolescents (1278 participants), from 25 German, Austrian, and Swiss centers specializing in pediatric obesity, revealed that elevated waist circumference, as well as BMI, are strongly correlated to parameters of hepatocyte injury—ALT and gammaglutamyl transferase (GGT) [31]. According to the recent scientific data, obese children with NAFLD diagnosed by both elevated serum ALT activity and liver steatosis on USG, have significantly higher BMI, WC values, ALT and GGT activities, HOMA-IR, and intensity of the hepatic steatosis in USG and intrahepatic lipid content in ^1^HMRS compared to other obese children without NAFLD [47]. Some studies also suggest that liver fat accumulation is a sensitive and early indicator of metabolic dysfunction [48]. This is in line with the results from the study of Papandreou et al., who demonstrated that the majority of children with hepatic steatosis assessed by USG fulfilled three or more criteria of MetS (58.6%) and showed significantly higher BMI, WC, TG, and lower HDL-C levels, as well as higher fasting insulin levels and higher IR assessed based on HOMA-IR, compared to patients with normal echogenicity of the liver. Moreover, in a logistic regression model, patients presenting with MetS have a three times higher risk of developing NAFLD compared to those without MetS [49]. Results from all these studies could be the reason for the necessity of undertaking routine control studies toward NAFLD in all obese patients and early lifestyle modifications to effectively reducing the overall risk of MetS. Additionally, in a recent study performed by Prokopowicz et al., a group of obese children with NAFLD was more likely to present MetS than those without NAFLD (40.82 versus 22.81%). NAFLD patients were characterized by greater WC, WHR, significantly higher serum concentration of TC, TG, fasting insulin, as well as glucose and insulin in 120 min of Oral Glucose Tolerance Test (OGTT). On the other hand, among the MetS components only hypertriglyceridemia was most often recognized in NAFLD patients than in those without NAFLD. Values of other components did not differ significantly in these group. Important here is the fact that only fasting glucose, but not insulin resistance, is considered in the IDF criteria for MetS. In these studies, NAFLD was significantly more often diagnosed in patients with HOMA-IR exceeding reference values than in children with the normal range of HOMA-IR (79 versus 28% for HOMA-IR > 90 percentile; 85 versus 15% for HOMA-IR > 97 percentile). According to the authors, HOMA-IR > 4.089 is a good indicator of NAFLD (AUROC (Area Under the Receiver Operating Characteristic) = 0.817, sensitivity = 70.8%, specificity = 83.6%) [50].

It has been proven that IR, which is described as a reduced effect of insulin in its target tissue, is increased in obese patients. Within the liver this will be manifested by reduced suppression of hepatic glucose production along with an increase in de-novo lipogenesis and very-low-density lipoprotein (VLDL) production. Normative values of IR as estimated by HOMA-IR calculated from fasting glucose and insulin concentrations. Diagnostic cutoffs of cardiometabolic risk factors in the pediatric population have been proposed by Shashaj et.al, who also included separate standards for obese children. Authors of this study observed that HOMA-IR > 3.42 in obese children is associated with an increased cardiometabolic risk, defined as a presence of at least one of the following: hypercholesterolemia, hypertriglyceridemia, reduced HDL-C levels, and ALT ≥ 40 U/L [51]. Patton et al., by recruiting 254 children and adolescents aged 6–17 years, have demonstrated that the severity of IR was significantly correlated with histological features of NAFLD and the risk of MetS was greater among those with severe steatosis [52]. While, Newton KP et al. evaluated the prevalence of T2DM and prediabetes in a large multi-center cohort of children with biopsy-proven NAFLD from pediatric centers across the United States. The researchers found that nearly 30% of children with NAFLD had abnormal glucose metabolism with 6.5% fulfill the criteria for T2DM. Notably, independent of age and BMI, girls with NAFLD were more likely to have T2DM than boys with NAFLD. Moreover, children with T2DM had a greater risk of having NASH (43.2%), regarded as a more progressive form of NAFLD, compared with prediabetes (34.2%) or those with normal glucose (22%) [53]. Similar observations were included in other studies, where T2DM was associated with more advanced forms of NAFLD within the studied pediatric and adult population [54,55]. Therefore, as one would expect, early detection of liver steatosis in high-risk populations is important for avoiding further development of severe forms of NAFLD. A recent study performed by Koutny et al. included obese patients aged 2–20 years from 51 centers, aimed to determine the prevalence of prediabetes and T2DM in patients with the increased and normal activity of ALT. They demonstrated that obese patients with mild (>24 to ≤50 U/L) or significant (>50 U/L) increase in ALT had greater odds ratios for prediabetes, while those with a significant increase in ALT also for T2DM compared to obese controls with normal liver transaminases activity. These findings may suggest that youth with NAFLD have a substantially higher risk of T2DM than obese youth in general [56].

As shown, IR represents a key factor linking the development of MetS features in children with liver steatosis. The strict connection between fatty liver and impaired insulin sensitivity has been documented, but considering the complexity of links between NAFLD, IR, and T2DM, it is extremely difficult to find out whether NAFLD is the effect or the cause of IR. For many years, NAFLD has been treated as the hepatic consequence of peripheral IR, which may affect the liver by different mechanisms [57]. It is most often explained by promoting intrahepatic lipid accumulation by up-regulation of free fatty acid (FFA) influx from adipocytes to hepatocytes. Excess of FFA in the portal system comes from accelerated by IR lipolysis of visceral fat and it may lead to fatty liver and MetS in different ways according to the genetic and epigenetic background. Furthermore, the consequence of this condition is upregulation of de novo lipogenesis, an impairment of FFA β-oxidation and over-secretion of VLDL, with a further increase in hepatic lipid accumulation [58,59,60]. Additionally, this excessive accumulation of lipid in hepatocytes may interfere with the normal insulin signal transduction pathway [61]. It is also well-established that altered production of adipose tissue-derived circulating factors called adipocytokines is also closely associated not only with obesity, but also with NAFLD and MetS [62,63,64,65,66]. The FFA overload in the hepatocytes together with the release of adipokines and proinflammatory cytokines coming from the adipose tissue are responsible for lipotoxicity, with a consequent mitochondrial dysfunction and increase in cytotoxic reactive oxygen species (ROS) production and the development of inflammatory response, predisposing to progressive liver damage [67].

However, recent evidence shows that the relation between NAFLD and components of metabolic syndrome is much more complicated and bi-directional. Based on recent research the theory that hepatic steatosis may precede IR and MetS, and even be a risk factor for their development, cannot be excluded, leading to possible further NAFLD exacerbation followed by progression towards NASH and fibrosis [16,68,69,70]. Assuming that NAFLD is the main cause of IR and MetS, it can be expected that therapies targeted at reducing liver fat storage may be successful in coping with the above metabolic disorders. Indeed, multiple studies across the world have revealed that lifestyle modification through changes in diet and physical activity (inducing a weight loss) in obese patients with NAFLD, apart from the beneficial effect on hepatic features, improved also insulin sensitivity and normalize several components of the MetS [71].

## 4. From NAFLD to MAFLD

NAFLD is regarded as hepatic manifestation of MetS and it is strongly associated with metabolic abnormalities. Data obtained from quoted publications suggest that a large part of obese patients with NAFLD present with concomitant metabolic risk factors. Based on this knowledge, recently, international panel of experts have proposed the change of the terminology from NAFLD to MAFLD, which strictly corresponds with comorbidities [72]. New MAFLD definition underlines coexistence of hepatic steatosis and metabolic dysfunctions, what is better reflection in the relation to disease etiology and pathogenesis. In this document, there are proposed criteria for the diagnosis of MAFLD, which are based on detection of liver steatosis by imaging techniques, blood biomarkers or scores, or by liver histology in addition to one of the following three criteria: overweigh or obesity, presence of T2DM, or evidence of metabolic dysregulation, characterized by the presence of at least two metabolic risk abnormalities (increased waist circumference, high blood pressure, hyperglyceridemia, reduced HDL-C concentration, glucose intolerance, or increase HbA1c, HOMA-IR ≥ 2,5, increased hs-CRP level in serum).

Recent data have shown that normal body weight does not protect from the development of NAFLD and it can also occur in non-obese individuals [73,74]. The incidence of lean NAFLD in adolescents ranging from 8% in the USA to 16% in the Asia–Pacific region [75]. According to MAFLD criteria, we are able to diagnose it also in non-overweight and non-obese patients, when we confirm a presence of liver steatosis with at least two mentioned above metabolic risk abnormalities. It should be noted that these non-obese patients with MAFLD are also at greater risk of liver damage and cardiovascular risk in comparison with metabolically healthy individuals [76].

The current NAFLD definition demands to rule out other causes of fatty liver. A new diagnostic strategy of MALFD, based on inclusion rather than exclusion criteria, accepts that development of fatty liver is associated with “multiple hits” and several different components may interact synergistically with metabolic factors [77,78]. Thus, MAFLD is a more specific term that describes more realistically the basis of this disease, especially multifactorial and metabolic-related pathophysiology of fatty liver disease, whereas current nomenclature with term “non” does not highlight the primary role of metabolic dysfunction in its pathogenesis and as a result it diminishes the importance of this condition. It can result in less involvement in seeking healthcare by patients. In fact, majority patients with NAFLD have a poor understanding of the disease. Even among medical practitioners NAFLD is undervalued, what can result in under-recognition and diagnosing accidentally at an advanced stage of disease.

**Figure 2:**
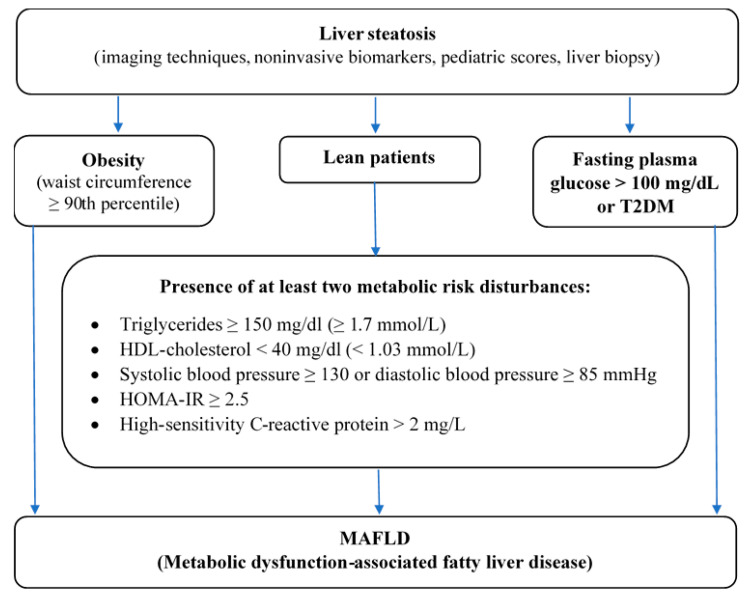
A proposed diagnostic criteria for MAFLD in patients aged 10–16 years old (adopted from Eslam et al. and IDF (International Diabetes Federation) definition of MetS in children and adolescents).

MAFLD diagnostic criteria have been developed exclusively for adults so far, however this new nomenclature could be also used successfully among children and adolescents, just to emphasize the relationship of fatty liver and metabolic disorders, which affect people independently of age. Hence, we come up with a proposal criteria set for the diagnosis of MAFLD in children and adolescents. According to our best knowledge this nomenclature in pediatric populations has not been introduced so far. In accordance with the IDF consensus report [37], which assumes that in the youngest children below the age of 10 years the MetS should not be diagnosed (however in these group weight reduction is highly recommended), it is possible to use this establishment in new MAFLD criteria. In younger children fatty liver disease is rather uncommon and often genetically determined in the course of various congenital metabolic diseases (e.g., cystic fibrosis, alfa1-antitrypsin deficiency, galactosemia, fructosemia, tyrosinemia type 1, mitochondrial acid peroxisomal defects of fatty acid oxidation) [79,80,81], therefore the diagnosis of MAFLD in the youngest group of patients seems to be rather rare. In the older children between 10 and 16 years old a diagnosis of MAFLD can be made based on the presence of hepatic steatosis in combination with one of the following three criteria:

1. Abdominal obesity assessed by WC ≥ 90th percentile adjusted for age and gender,
2. High fasting plasma glucose > 100 mg/dl or known T2DM or
3. Presence of at least two metabolic risk disturbances in lean patients—elevated triglycerides, low HDL-C, high blood pressure, HOMA-IR ≥ 2,5, increased hs-CRP (Figure 2).

MAFLD adult criteria can be used for adolescents aged more than 16 years old. The proposed redefinition of the disease has the potential to improve people’s awareness about the pathogenesis of this common liver condition and help in understanding multiple metabolic dysfunctions that affect the human body from an early age. Moreover, it holds a message that proper lifestyle modification, which leads to weight reduction, has a beneficial influence on both metabolic and hepatic features in these patients. Furthermore, proposed redefinition of the disease, as well as taking into consideration the greater impact of metabolic dysfunction on liver condition, could facilitate the identification of this kind of patient. Nowadays there is a huge development of new, noninvasive markers useful for the prediction of hepatic steatosis, and progression to steatohepatitis and hepatic fibrosis, without the need for routine performing of liver biopsy [82]. Hence, there is a great chance to improve discovering new non-invasive predictors for MAFLD. Finally, earlier diagnosis of fatty liver is synonymous with sooner intervention and fewer complications in future life.

Although, the natural history of MAFLD in children is not thoroughly known, we speculate that accelerated progression of liver disease and metabolic disturbances can increase the cardiovascular risk, although further investigation in this area are needed. With our understanding of the disease etiology and pathogenesis, new term “MAFLD” characterize the disease more fully and it seems to be more suitable for guiding clinical practice and scientific research.
